# Use of bacteriophages in the treatment of colistin-only-sensitive *Pseudomonas aeruginosa* septicaemia in a patient with acute kidney injury—a case report

**DOI:** 10.1186/s13054-017-1709-y

**Published:** 2017-06-04

**Authors:** Serge Jennes, Maia Merabishvili, Patrick Soentjens, Kim Win Pang, Thomas Rose, Elkana Keersebilck, Olivier Soete, Pierre-Michel François, Simona Teodorescu, Gunther Verween, Gilbert Verbeken, Daniel De Vos, Jean-Paul Pirnay

**Affiliations:** 1 Burn wound center, Queen Astrid military hospital, Brussels, Belgium; 2 Laboratory for molecular and cellular technology, Queen Astrid military hospital, Brussels, Belgium; 3 Phage therapy center, Queen Astrid military hospital, Brussels, Belgium

**Keywords:** *Pseudomonas aeruginosa*, Antibiotic resistance, Colistin, Bacteraemia, Acute kidney injury, Intravenous, Bacteriophage therapy

Sepsis from *Pseudomonas aeruginosa* bacteraemia may be fatal, especially when no appropriate therapy can be given.

In June 2016, a 61-year-old man was hospitalised for *Enterobacter cloacae* peritonitis and severe abdominal sepsis with disseminated intravascular coagulation, secondary to a diaphragmatic hernia with bowel strangulation. The patient had a prolonged hospital course complicated by gangrene of the peripheral extremities, resulting in the amputation of the lower limbs and the development of large necrotic pressure sores (Fig. 1).

**Fig. 1:**
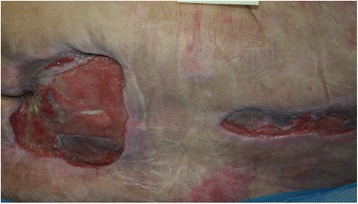
Large pressure sores in the sacral and spinal back areas. Situation on 17 November 2016

Three months after admission, the patient was transferred to the Queen Astrid military hospital for surgical management of the pressure sores. Wound cultures on admission revealed colonisation with, amongst others, multidrug-resistant *P. aeruginosa*. One month after admission, the patient developed septicaemia with colistin-only-sensitive *P. aeruginosa*. Intravenous colistin therapy was started.

Ten days later, the patient developed acute kidney injury with rising serum creatinine and urea levels (Fig. 2), presumably sepsis- and drug-induced. Antibiotic therapy was discontinued to prevent further kidney damage. Unfortunately, on 12 November, *P. aeruginosa* septicaemia re-emerged with rapid heart rate, low blood pressure, fever and high C-reactive protein (CRP) levels. On 14 November, the patient went into a coma with 9 scores (E2V2M5) on the Glasgow Coma Scale. Because of the risk of colistin nephrotoxicity and the family’s will to avoid intensive therapy interventions such as hemofiltration, bacteriophage therapy was initiated under the umbrella of Article 37 (Unproven Interventions in Clinical Practice) of the Declaration of Helsinki [1]. Fifty microlitres of purified bacteriophage cocktail BFC1 [2], containing two bacteriophages that showed in vitro activity against the patient’s *P. aeruginosa* isolates, were administered as a 6-h intravenous infusion for 10 days. Wounds were irrigated with 50 ml BFC1 every 8 h for 10 days.

**Fig. 2:**
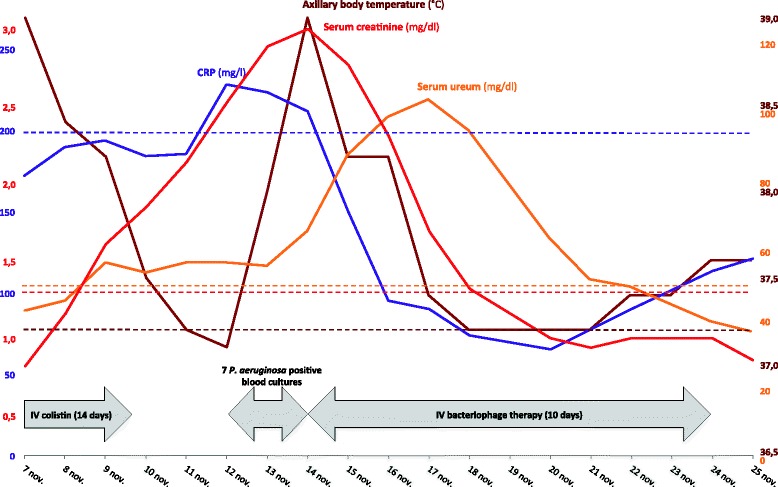
Timeline showing relevant clinical parameters and treatments. *Dotted lines* mark the upper threshold values. *CRP* C-reactive protein

Immediately, blood cultures turned negative, CRP levels dropped and the fever disappeared (Fig. 2). Kidney function recovered after a few days (Fig. 2). Hemofiltration was avoided and no unexpected adverse events, clinical abnormalities or changes in laboratory test results that could be related to the application of bacteriophages were observed. The pressure sores remained infected with several bacterial species, including *P. aeruginosa*, causing multiple episodes of sepsis, which were treated using empirical antibiotic therapy. Unfortunately, the patient died 4 months after bacteriophage therapy of sudden in-hospital refractory cardiac arrest due to blood culture-confirmed *Klebsiella pneumoniae* sepsis. In vitro susceptibility testing revealed that the *K. pneumoniae* strain was sensitive to the antibiotics the patient was on.

Bacteriophages are increasingly put forward as safe alternatives or additions to antibiotic therapy. Historical reports show that they were efficaciously used via the intravenous route, especially in typhoid fever and *Staphylococcus aureus* bacteremia [3], but this is—as far as we know—the first contemporary report of intravenous bacteriophage monotherapy against *P. aeruginosa* septicaemia in humans.

## Abbreviations

CRP: C-reactive protein
IV: intravenous
